# Optimization of process conditions for tannin content reduction in cassava leaves during solid state fermentation using *Saccharomyces cerevisiae*

**DOI:** 10.1016/j.heliyon.2019.e02298

**Published:** 2019-08-19

**Authors:** Mohamed Hawashi, Ali Altway, Tri Widjaja, Setiyo Gunawan

**Affiliations:** Department of Chemical Engineering, Institut Teknologi Sepuluh Nopember (ITS), Surabaya, 60111, Indonesia

**Keywords:** Bioengineering, Chemical engineering, Biochemistry, Cassava leaves, Optimization, Response surface methodology, Solid state fermentation, Tannin content, Tannase, Biochemical engineering, Transport process, Industrial chemistry, Materials characterization, Degradation

## Abstract

Cassava leaves are a crucial source of alternative protein resources for both humans and livestock in developing societies in African and Asian countries that do not have easy access to available protein sources. Hence, cassava has the capacity to promote the economic development of these countries and provide food security. However, it has some disadvantages due to the anti-nutrient compounds present in its tissues, which limits the nutritional value of cassava leaves. Thus, proper processing of cassava leaves is essential in order to reduce the anti-nutrients to a safer limit before utilization. This study focuses on reducing the tannin content of cassava leaves during solid-state fermentation using *Saccharomyces cerevisiae*. In addition, the Box-Behnken design of the Response Surface Methodology was applied to optimize various process parameters, such as carbon concentration, nitrogen concentration, moisture content, and incubation time for maximum reduction of tannin content in cassava leaves. A quadratic model was developed for the reduction of tannin content, which resulted in a perfect fit of the experimental data (p < 0.01). The optimal conditions were found at 1.4% (w/w) of carbon concentration, 0.55% (w/w) of nitrogen concentration, 57% (v/w) moisture content, and an incubation time of 96 h. The minimum tannin content obtained under these conditions was 0.125%, which indicated a reduction of 89.32 % in tannin content. Conversely, the protein content was increased with a further increase in fermentation time from 24 to 96 h (from 10.08 to 14.11–16.07 %). Furthermore, the ability of *Saccharomyces cerevisiae* to produce tannase under solid-state fermentation of cassava leaves was also studied. The maximum yield was obtained with an enzyme activity of 0.53 U/gds after 72 h of incubation.

## 1 Introduction

Cassava plant (*Manihot esculenta*) is grown throughout the tropics for its roots as an important carbohydrate source (Gunawan et al., 2015). The production of cassava roots, which have a comparable yield with the leaves, is estimated at 10 tons per hectare (Morgan and Choct, 2016). Cassava leaves have been identified as a rich source of protein, minerals, and vitamins that exceed some of the other green vegetables and foodstuff (Wargiono et al., 2011; Pereira et al., 2016). The consumption of cassava leaves has increased significantly in Indonesia and is estimated to be around 0.5–0.7 million tons per year (Wargiono et al., 2011). However, cassava leaves also have certain disadvantages due to the toxic compounds (e.g., cyanogenic glycoside) and are anti-nutrients (e.g., phytic acid, tannins, and polyphenols) in its tissues (Fasuyi, 2005; Latif and Müller, 2015). The proportions of these compounds are mostly dependent on cultivars of cassava. Anti-nutrients are compounds that act against digestibility and absorption of nutrients (Wobeto et al., 2007). The over-consumption of anti-nutrient rich food may lead to adverse effects, such as poisoning, depending on the amount consumed and the treatment method available (Bradbury and Nixon, 1998; Montagnac et al., 2009). At a tolerable amount, it may bring benefits to human due to their antioxidant properties (Wobeto et al., 2007).

Among the anti-nutrients, tannin is a high-molecular-weight compound consisting of phenolic hydroxyl groups that forms strong complexes with protein (Soest et al., 1987). The condensed tannins found in cassava leaves play a harmful role, decreasing the protein digestibility (Reed et al., 1982). Aside from functioning as an antioxidant, polyphenols are capable of binding essential minerals whose absorption will be affected (Latif and Müller, 2015). As for the cassava leaves, polyphenols are in the form of condensed tannins consisting of anthocyanidins, particularly as cyanidin and delphinidin (Padmaja, 1989). Consequently, proper processing of cassava leaves is essential to diminish the toxic compounds and anti-nutrients to a safe limit for human utilization.

In recent years, solid-state fermentation (SSF) has been applied as an economical and efficient processing method for enrichment and detoxifying the cassava components (Behera and Ray, 2017).

So far, only a few studies on the fermentation of cassava leaves are available while most of these studies investigate the root as the raw material. Among the efforts made, Morales et al. (2018) investigated the efficiency of SSF with *Rhizopus oligosporus* in reducing cyanide content and improving the nutritional value of cassava leaves.

Microorganisms play an important role in producing multiple microbial enzymes during SSF, such as tannase, which is responsible for hydrolyzing tannic acid to give glucose and gallic acid (Sabu et al., 2006; Liu et al., 2016).

*Saccharomyces cerevisiae* has been widely used in traditional fermentation due to several advantages, such as the ability to secrete extracellular enzymes, non pathogenicity, and low cost (Nasseri et al., 2011; Aruna et al., 2017).

Environmental factors play a decisive role in the SSF process and can affect microbial growth and product formation (Raimbault, 1998; Manpreet et al., 2005). The process used for optimisation using the One Variable at One Time (OVAT) method (changing one single variable while other variables are kept constant) does not work properly, since it is costly and requires several experiments to achieve optimum levels (Shojaosadati et al., 1999; Nygaard et al., 2019).

Response Surface Methodology (RSM) is a useful application to optimize the process conditions and evaluate the correlation between independent variables and their responses (Rana et al., 2012; Ruqayyah et al., 2014). The use of RSM to optimize the fermentation system has several advantages: (i) reduces the number of process parameters, (ii) evaluates the interaction between variables and responses, and (iii) offers cost-effective design (Shaktimay et al., 2010; Jamal et al., 2012).

Therefore, the main aim of this study was to determine the optimum conditions for the solid-state fermentation with *S. cerevisiae* using response surface methodology (RSM) to minimize the tannin content in cassava leaves. Chemical composition and tannase production as a function of cassava leaves processing were also evaluated under optimal SSF conditions.

## 2 Materials and methods

### 2.1 Materials

Cassava leaves (Litbang UK-2) were obtained from a cassava plantation in Menganti, Gresik. The *S. Cerevisiae* strain (InaCCY655) was obtained from the Research Center for Biology, Indonesian Institute of Sciences (LIPI).

### 2.2 Inoculum preparation

The strain of *S. cerevisiae* was cultured in a 500 mL Erlenmeyer culture flask containing 100 mL of Potato Dextrose Broth (PDB) medium. The yeast strain was then incubated in a shaking incubator at 30 °C and 200 rpm for 24 h until the yeast cell density of 1 (OD_600nm_) was reached.

### 2.3 Determination of tannin content

The tannin content of the unfermented and fermented leaves was measured using the method described by Makkar and Goodchild (1995). Briefly, 200 mg of each ground dried leaves were transferred into a conical flask containing 10 mL of aqueous acetone solution. The solution containing the leaves was thoroughly soaked for 15 h. The tannin was collected as a supernatant in a flask using a filter paper. Immediately, 6 mL of butanol-HCl reagent was added to 1 mL of extract in a test tube. The tube was heated at 120 °C using a dry block heater (QBH2 Biophoretics, USA) for 1 h and then cooled at ambient temperature. The absorption was then read at 550 nm with a spectrophotometer (Thermo scientific evolution 160 UV-VIS).

### 2.4 Chemical composition analysis

The chemical composition (crude protein, ash, moisture content, crude fiber, carbohydrate) of unfermented and fermented cassava leaves was determined using standard analytical procedures of AOAC (2005). Fat content was determined as described in previous work (Gunawan et al., 2013). The results were expressed as % dry weight basis (dw).

### 2.5 Enzyme assay

An incubation time of four days was used for each experiment, and samples were taken every 24 h. The crude enzymes from the fermented leaves were extracted by using 50 mL of 0.05 M citrate buffer solution (pH = 5.0). The filtrates were collected in a 15-mL centrifuge tube using filter paper. The extracted solution was centrifuged at 12,000 × g for 30 min. The resulting supernatant was used as a source of crude enzyme. The activity of tannase was measured using the method described by Sharma et al. (2000). The determination of tannase activity is evaluated based on the formation of a chromogen between gallic acid (which is released by methyl gallate action) and rhodanine. The tannase activity was defined in unit per gram of dry-based substrate (U/gds).

### 2.6 Solid-state fermentation (SSF)

The substrate containing 50 g of dried cassava leaves were weighed in a 500 mL Erlenmeyer flask. Carbon sources at 1, 2, and 3% (w/w) and nitrogen sources at 0.5, 1, and 1.5% (w/w) were added to the substrate in the form of sucrose and urea, respectively. The moisture content of the mixtures was adjusted to 45, 60, and 75% (v/w) by adding 50, 75, and 150 mL of distilled water, respectively. The Erlenmeyer flasks containing the mixtures were sterilized at 121 °C for 10 min then cooled at 26 °C and kept for 30 min. Next, 14 mL (1.9 × 10^8^ CFU/g) of inoculum was added to each flask. Cotton and aluminum foils were used to cover the flasks. The mixture was fermented at 30 °C for different periods (24, 60, and 96 h).

### 2.7 Experimental design and statistical analysis

In the current research, a Box-Behnken design of RSM with four independent variables, which includes carbon concentration (A), nitrogen concentration (B), moisture content (C), and incubation time (D) was used. The Box-Behnken design consisted of 29 experimental runs with each independent variable being at three levels (−1, 0, and 1), as listed in Table 1. The independent variables were coded by Eqs. (1), (2), (3), and (4).

(1) $$A=\frac{\left(Carbonconcentration-2\right)}{1}$$

(2) $$B=\frac{\left(Nitrogenconcentration-1\right)}{0.5}$$

(3) $$C=\frac{\left(Moisturecontent-60\right)}{15}$$

(4) $$D=\frac{\left(Incubationtime-60\right)}{36}$$

**Table 1:** The level of the independent variables used in RSM.

Independent variables	Unit	Code	Actual levels of coded factor		
			-1	0	+1
Carbon concentration	%	A	1	2	3
Nitrogen concentration	%	B	0.5	1	1.5
Moisture content	%	C	45	60	75
Incubation time	h	D	24	60	96

The experimental data were fitted by using the second-order polynomial equation shown in Eq. (5).

(5) $$Y=\beta_{0}+\beta_{1}A+\beta_{2}B+\beta_{3}C+\beta_{4}D+\beta_{11}A^{2}+\beta_{22}B^{2}+\beta_{33}C^{2}+\beta_{44}D^{2}+\beta_{12}AB+\beta_{13}AC+\beta_{14}AD+\beta_{23}BC+\beta_{24}BD+\beta_{34}CD+\varepsilon$$

where, Y is the response; β_0_ is the equation constant; β_2_, β_3,_ and β_4_ are the linear coefficients; β_11_, β_22_, β_33_, and β_44_ are the quadratic coefficients; β_12_, β_13_, β_14_, β_23_, β_24_, and β_34_ are the interaction coefficients; and ε is the error term.

(6) Error%=(Experimentalvalue–Predictedvalue)/Experimentalvalue×100

The error between the predicted and the actual values were calculated from Eq. (6).

The data were analyzed using the analysis of variance (ANOVA) in order to estimate the F value, lack of fit, and determination coefficient (R^2^) of the experimental model, as well as the effects of linear, interaction, and quadratic terms on tannin content. RSM and analysis of data were conducted by the Design-Expert software (Version 11.0, Stat-Ease, USA).

## 3 Results and discussion

### 3.1 Tannin reduction during solid-state fermentation

The experimental data for tannin content in the processed leaves with various SSF conditions are given in Table 2. The residual levels of tannin content in fermented leaves ranged from 0.15 to 0.85%. The initial tannin content of the dried cassava leaves was of 1.17%. After 60 h of fermentation with *S. cerevisiae*, the residual tannin content in the fermented leaves was lower than the undesirable tannin level (0.7–0.9%) (Aletor, 1993). These results are in accordance to a study performed by Oboh and Elusiyan (2007), which showed a remarkable decrease in tannin content of cassava flour (75% removal) under SSF using *S. cerevisiae* for 72 h. These results showed the high effectiveness of the solid-state fermentation relative to tannin removal compared to the conventional methods of steaming (STM) and oven-drying (ODV). The STM method (above the boiling point of water for 30 min) or ODV method (80–90 °C for 24 h) of cassava leaves were inefficient and could only yield to 22–36% removal of tannin content (Fasuyi, 2005).

**Table 2:** Experimental design of Box-Behnken.

Run No	A: Carbon Concentration (%)	B: Nitrogen Concentration (%)	C: Moisture Content (%)	D: Incubation Time (h)	Response Tannin (% dw)
1	2 (0)	0.5 (-1)	60 (0)	96 (1)	0.15
2	1 (-1)	1.5 (1)	60 (0)	60 (0)	0.44
3	2 (0)	1.5 (1)	45 (-1)	60 (0)	0.55
4	3 (1)	1 (0)	60 (0)	96 (1)	0.21
5	1 (-1)	1 (0)	60 (0)	96 (1)	0.17
6	2 (0)	1.5 (1)	75 (1)	60 (0)	0.53
7	3 (1)	0.5 (-1)	60 (0)	60 (0)	0.41
8	2 (0)	1.5 (1)	60 (0)	96 (1)	0.19
9	1 (-1)	0.5 (-1)	60 (0)	60 (0)	0.29
10	2 (0)	1 (0)	60 (0)	60 (0)	0.37
11	3 (1)	1 (0)	60 (0)	24 (-1)	0.80
12	2 (0)	1.5 (1)	60 (0)	24 (-1)	0.79
13	2 (0)	1 (0)	45 (-1)	96 (1)	0.26
14	3 (1)	1 (0)	75 (1)	60 (0)	0.54
15	2 (0)	1 (0)	75 (1)	96 (1)	0.23
16	1 (-1)	1 (0)	60 (0)	24 (-1)	0.76
17	2 (0)	1 (0)	60 (0)	60 (0)	0.39
18	2 (0)	1 (0)	45 (-1)	24 (-1)	0.85
19	2 (0)	0.5 (-1)	45 (-1)	60 (0)	0.46
20	2 (0)	1 (0)	60 (0)	60 (0)	0.38
21	3 (1)	1 (0)	45 (-1)	60 (0)	0.57
22	1 (-1)	1 (0)	45 (-1)	60 (0)	0.51
23	2 (0)	1 (0)	60 (0)	60 (0)	0.35
24	1 (-1)	1 (0)	75 (1)	60 (0)	0.43
25	3 (1)	1.5 (1)	60 (0)	60 (0)	0.45
26	2 (0)	0.5 (-1)	60 (0)	24 (-1)	0.78
27	2 (0)	1 (0)	60 (0)	60 (0)	0.36
28	2 (0)	0.5 (-1)	75 (1)	60 (0)	0.42
29	2 (0)	1 (0)	75 (1)	24 (-1)	0.83

### 3.2 Model development

Statistical tests were used to evaluate the appropriateness of experimental data to the mathematical models. The best model was chosen based on the highest R^2^, adjusted R^2^, and predicted R^2^ values. In line with these values, the quadratic model was the most suitable to explain the experimental data. The values of the statistical parameters calculated for all proposed models are summarized in Table 3.

**Table 3:** Statistical result for the mathematical models.

Model	Lack of fit (p-value)	Sequential p-value	Adjusted R^2^	Predicted R^2^	R^2^	Remarks
Linear	0.0042	<0.0001	0.8937	0.8756	0.9089	
2FI	0.0023	0.9939	0.8633	0.7921	0.9121	
Quadratic	0.1359	< 0.0001	0.9852	0.9609	0.9926	Suggested
Cubic	0.0758	0.4603	0.9861	0.6888	0.9970	Aliased

2FI is two-factor interaction model.

### 3.3 Response surface analysis

The appropriateness of the developed model was checked by ANOVA. As indicated by the obtained ANOVA results (Table 4), an F-value of 134.47 indicates that the model was significant at p < 0.0001 and the probability that F-value is due to noise was only 0.01%. According to Ferreira et al. (2007), the comparison between the residual and the pure error is called lack of fit. The F-value obtained from the lack of fit was 3.21, which indicates that the lack of fit was insignificant (p > 0.05). Non-significant "lack of fit" was acceptable and therefore the number of experiments was sufficient to evaluate the effects of variables on the response (Silvestrini et al., 2013).

**Table 4:** Analysis of variance (ANOVA).

Source	Sum of square	df	Mean square	F-value	P-value
Model	1.21	14	0.0868	134.47	<0.0001
A	0.0120	1	0.0120	18.65	0.0007
B	0.0161	1	0.0161	25.00	0.0002
C	0.0040	1	0.0040	6.25	0.0255
D	1.08	1	1.08	1673.80	<0.0001
AB	0.0030	1	0.0030	4.69	0.0481
AC	0.0006	1	0.0006	0.9686	0.3417
AD	2.220E-16	1	2.220E-16	3.441E-13	1.0000
BC	0.0001	1	0.0001	0.1550	0.6998
BD	0.0002	1	0.0002	0.3487	0.5643
CD	0.0000	1	0.0000	0.0387	0.8468
A^2^	0.0052	1	0.0052	8.07	0.0131
B^2^	0.0012	1	0.0012	1.79	0.2026
C^2^	0.0693	1	0.0693	107.34	<0.0001
D^2^	0.0450	1	0.0450	69.81	<0.0001
Residual	0.0090	14	0.0006		
Lack of Fit	0.0080	10	0.0008	3.21	0.1359
Pure error	0.0010	4	0.0003		

According to Ahirwar et al. (2016), the model with a low coefficient of variation value (CV) indicates the high reliability of the experiment. In this study, it has been proven that the model CV obtained was 5.47%, which showed both greater accuracy in experiments and greater reliability.

**Fig. 1:**
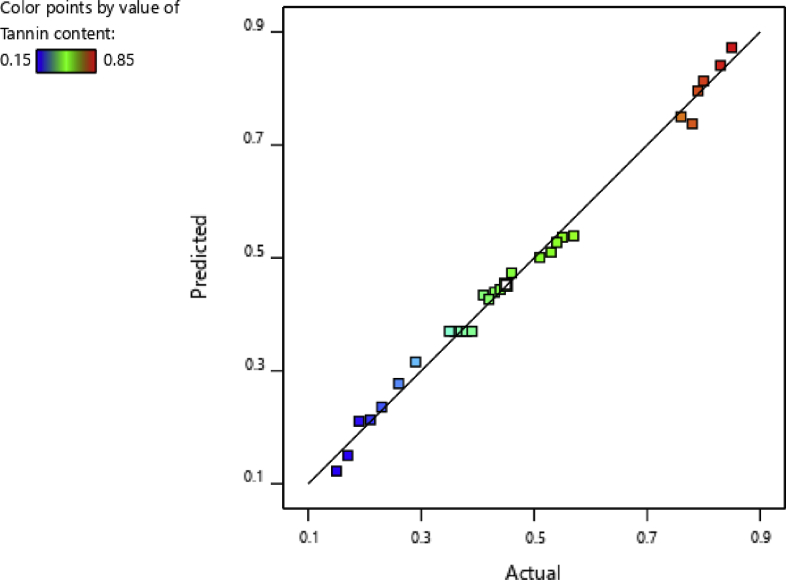
Predicted versus actual values for tannin content.

The determination coefficient (R^2^) of the model was 0.9926, suggesting that 99.26% of the experimental values of tannin content matched with the predicted values by the developed model (Fig. 1). Moreover, the predicted R^2^ of 0.9609 was reasonably consistent with the adjusted R^2^ of 0.9852. The adequate precision value measures the signal-to-noise ratio, including the expected values and the predicted average errors (Biswas et al., 2019). According to Isar et al. (2006), the recommended value of the adequacy precision ratio should be higher than 4. In this study, the value is 41,05, which was significantly higher than the recommended value. Therefore, the developed model is expected to predict the parameters and results with high accuracy. The effects of the independent variables on tannin content were evaluated by using Eq. (7).

(7) $$Tannin=+2.896-0.0766A-0.116B-0.0583C-0.0161D-0.0550AB+0.0008AC-4.3108AD+0.0006BC+0.0004BD-4.6296CD+0.0283A^{2}+0.0533B^{2}+0.0004C^{2}+0.0001D^{2}$$

The results of the statistical analysis indicated that the effects of incubation time (D) and quadratic model terms (C^2^ and D^2^) on tannin content were highly significant at p < 0.01. Also, the effects of A, B, C, A^2^, and AB were significant at p < 0.05. While the probability value (p-value) greater than 0.05 suggests that the terms of the model were insignificant.

Based on the regression of linear and quadratic coefficients, the order of independent variables affecting the tannin content was incubation time > moisture content > carbon concentration > nitrogen concentration.

### 3.4 The synergistic effects of the independent variables on the tannin content

**Fig. 2:**
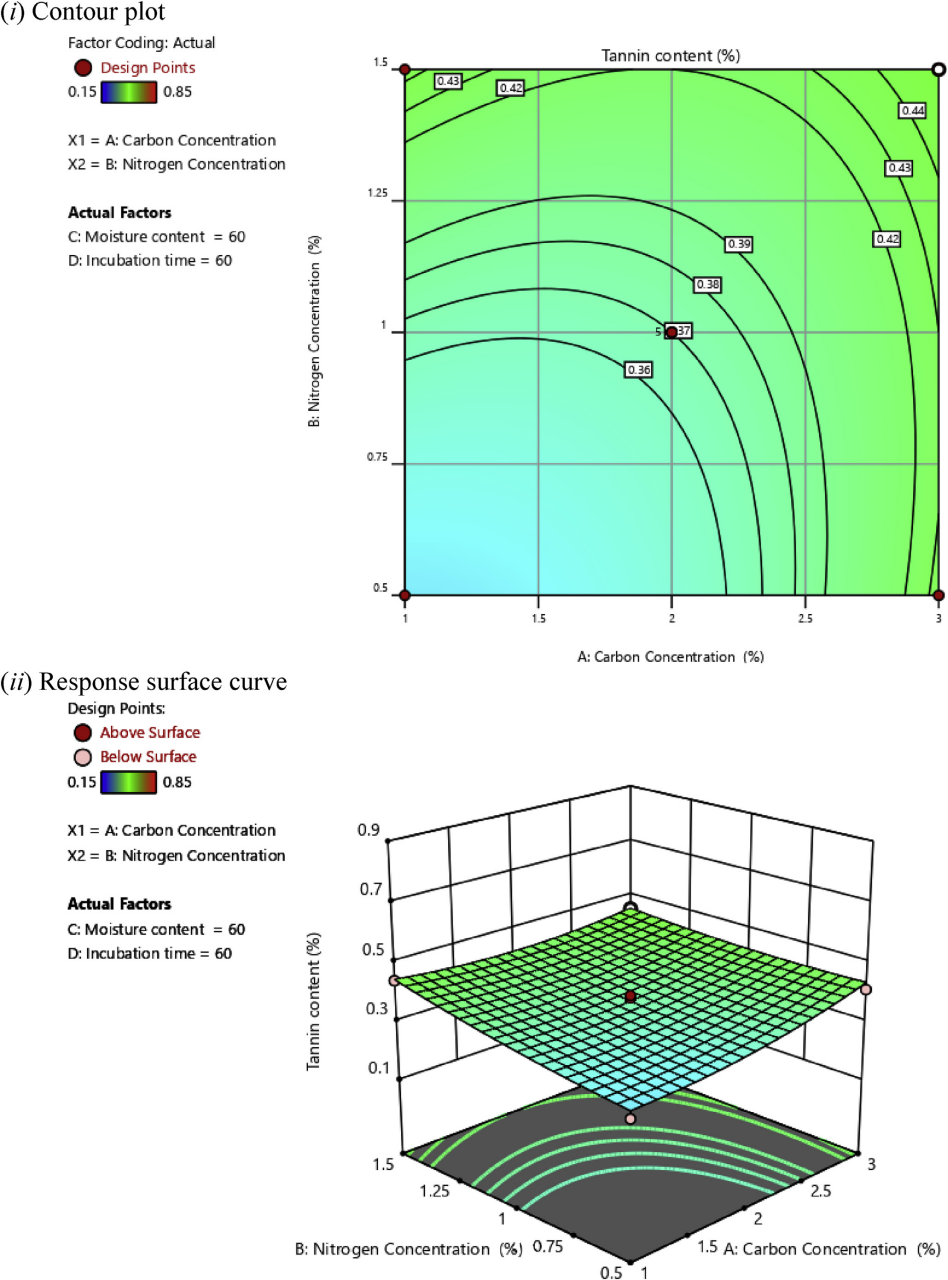
(*i*) Contour plot and (*ii*) response surface curve showing the interaction effect of carbon concentration and nitrogen concentration on the tannin content.

**Fig. 3:**
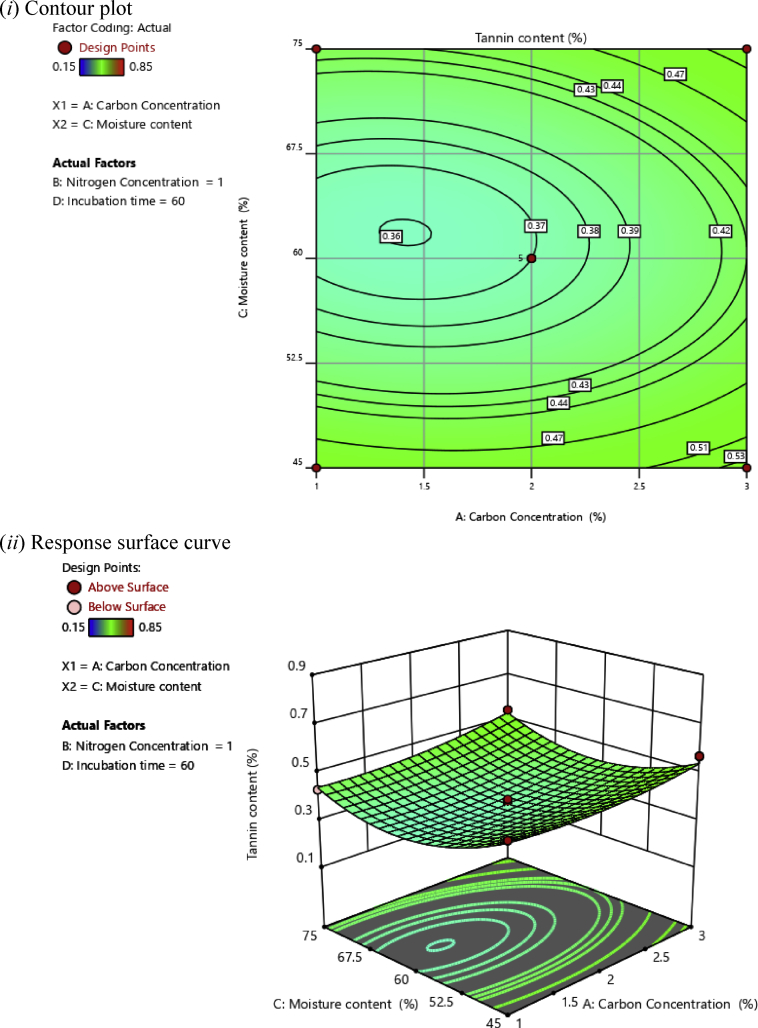
(*i*) Contour plot and (*ii*) response surface curve showing the interaction effect of carbon concentration and moisture content on the tannin content.

**Fig. 4:**
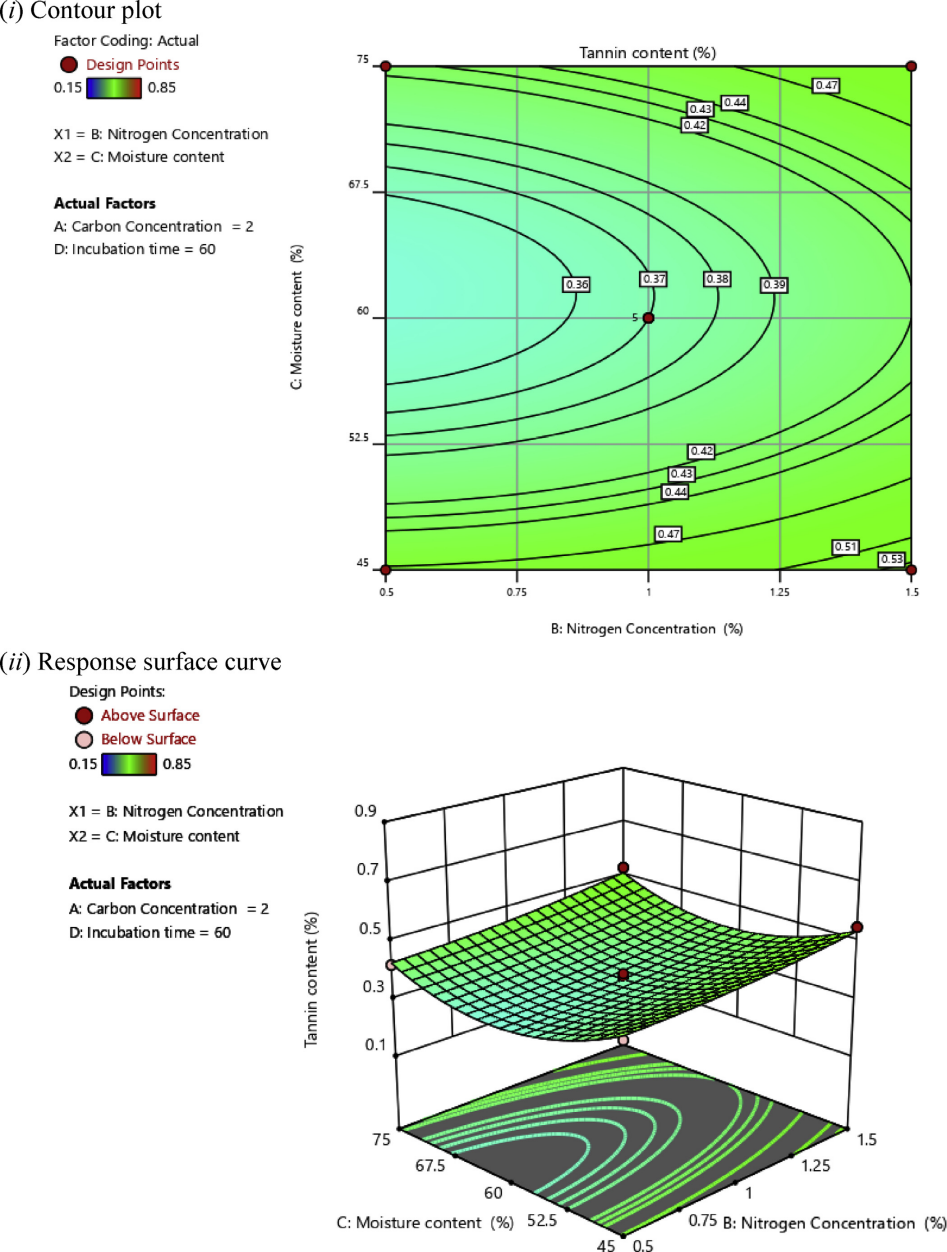
(*i*) Contour plot and (*ii*) response surface curve showing the interaction effect of nitrogen concentration and moisture content on the tannin content.

**Fig. 5:**
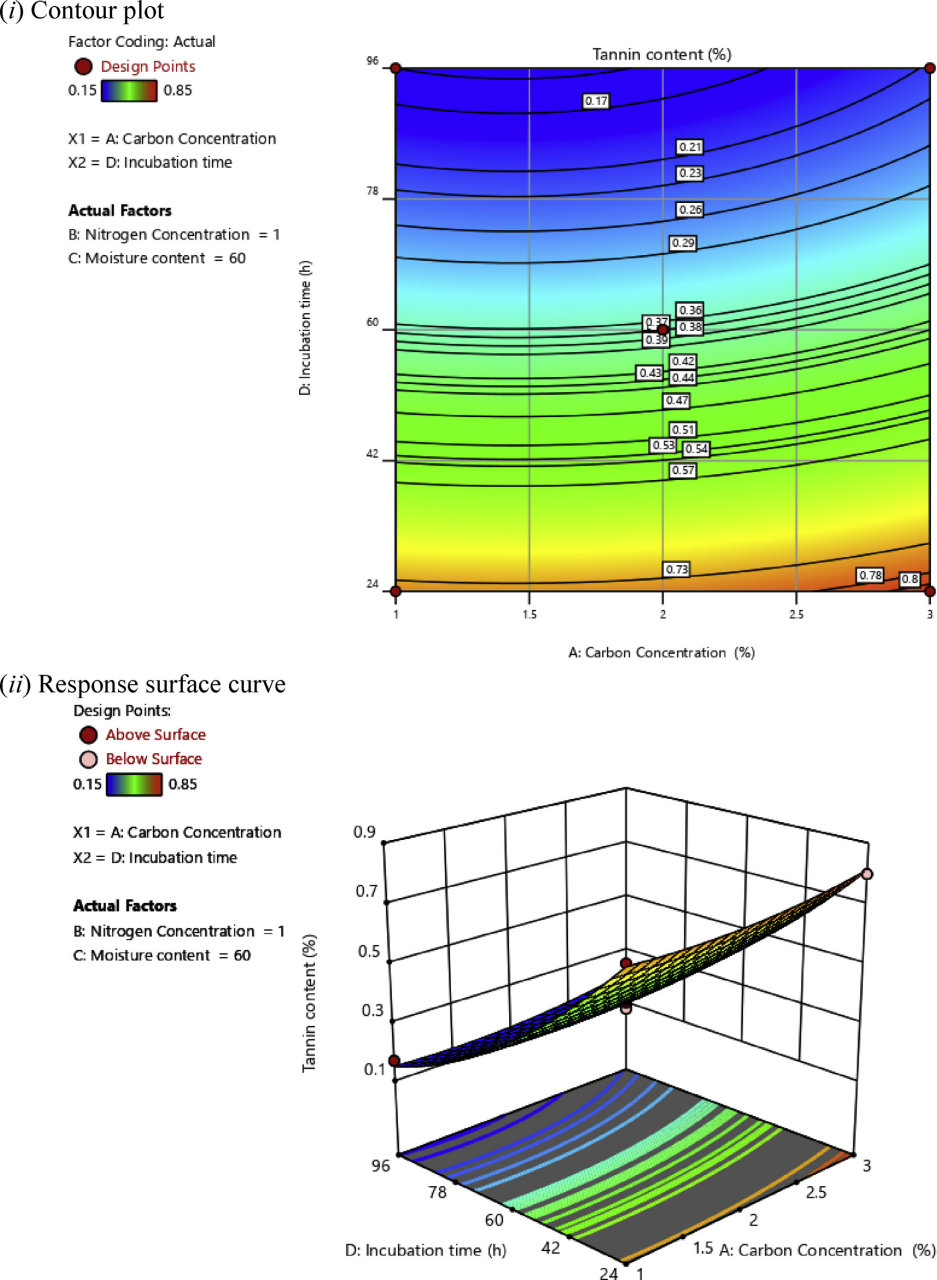
(*i*) Contour plot and (*ii*) response surface curve showing the interaction effect of carbon concentration and incubation time on the tannin content.

**Fig. 6:**
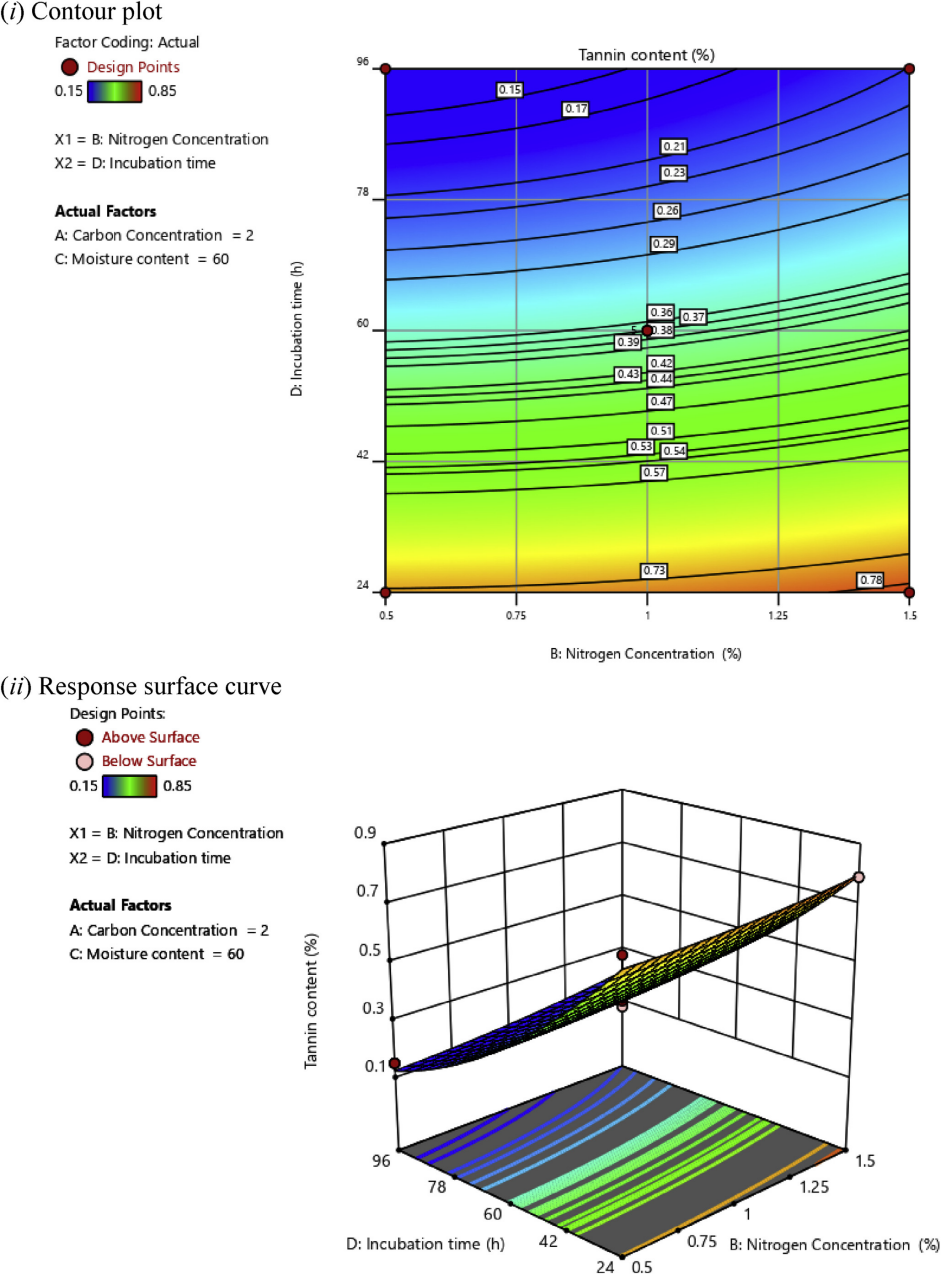
(*i*) Contour plot and (*ii*) response surface curve showing the interaction effect of nitrogen concentration and incubation time on the tannin content.

**Fig. 7:**
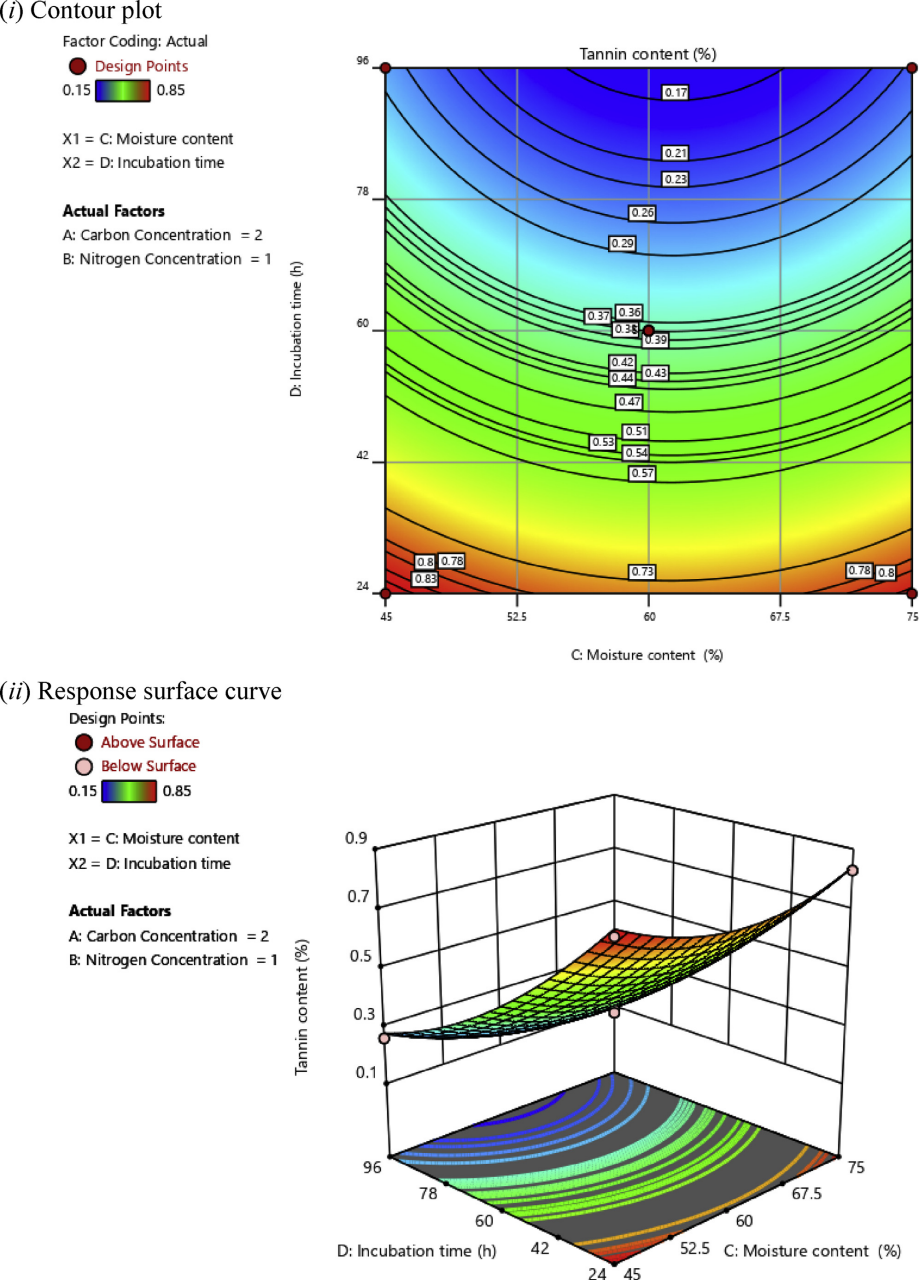
(*i*) Contour plot and (*ii*) response surface curve showing the interaction effect of moisture content and incubation time on the tannin content.

In this investigation, based on the polynomial regression equations, two-dimensional (2D) contour plots and three-dimensional (3D) response surface plots were drawn as a function of two independent variables while maintaining two other independent variables at an optimal level aiming at understanding the synergistic effects of factors on the tannin content of cassava leaves, as illustrated in Figs. 2, 3, 4, 5, 6, and 7.

Contour plots help to determine the level of variables that contribute to the desired response, whereas response surface plots help to determine the minimum, middle, and maximum response points (Ahirwar et al., 2016). The desirable synergistic effects between two independent variables have the elliptical shape of the response surface plots (Muralidhar et al., 2001).

Fig. 2 shows the synergistic effect of the carbon and nitrogen concentration on tannin content at a constant incubation time of 60 h and 60% moisture. It was observed that tannin content decreased for both the low concentration of carbon and nitrogen. The minimum tannin content obtained for the combined effect between carbon and nitrogen concentration was 35%. However, successful fermentation requires appropriate microbial activities, which are impacted by the quality and availability of carbon and nitrogen sources. Carbon and nitrogen are major components of vital biological structures (proteins, DNA/RNA, etc.) and as such carbon- and nitrogen-containing substrates are necessary to support microbial growth and proliferation (Gunawan et al., 2015).

Fig. 3 shows the synergistic effects of carbon concentration and moisture content on tannin content at a constant concentration of nitrogen of 1% and 60 h of incubation time. The tannin content increased with the increase in carbon concentration. On the contrary, the mid-level moisture content had a significant effect on the tannin content.

Fig. 4 shows the synergistic effects of nitrogen concentration and moisture content on tannin content at a constant concentration of 2% carbon and 60 h of incubation time. Higher tannin content was observed at higher levels of nitrogen concentration. On the contrary, the low nitrogen concentration had a more significant effect on the tannin content. The plot clearly shows that the minimum tannin content was obtained at mid-level humidity (60%).

Fig. 5 shows the synergistic effects of carbon concentration and incubation time at a constant moisture content of 60% and 1% nitrogen concentration. The minimum tannin content obtained was 0.17%. To note, in this case, the effect of incubation time was more significant than the concentration of carbon.

Fig. 6 shows the synergistic effects between nitrogen concentration and incubation time on tannin content with the optimum levels of carbon concentration and moisture content, i.e., 2 and 60%, respectively. The lowest tannin content (0.15%) was obtained at 0.5% nitrogen concentration and 96 h of incubation time. From these results, it was observed that the tannin content decreased significantly with the increase in incubation time.

Fig. 7 shows the synergistic effects of moisture content and incubation time on tannin content at a constant concentration of carbon and nitrogen of 2 and 1%, respectively.

In this study, it was observed that the tannin content increased in both higher and lower levels of moisture content. Previous researchers have observed similar trends in the SSF system. If the moisture level drops too low, the degree of swelling becomes low resulting in higher water tension while high moisture levels cause the porosity of the substrate to decrease, resulting in lower diffusion rates and lower gas exchange (Mustafa et al., 2016; Sondhi and Saini, 2019). The contour and surface plots revealed that the effect of the incubation time was more pronounced on tannin content. With prolonged incubation time, the content of tannin decreased until it reached 0.17%. The decrease in tannin content may have resulted from the hydrolysis catalyzed by tannase enzyme.

### 3.5 Process optimization and model validation

To determine the optimal conditions of the SSF process, the numerical optimization method by using Design-Expert software was applied. Table 5 shows the predicted optimal SSF conditions for minimizing the tannin content. The tannin content predicted at optimum level was 0.122%. Three experiments were conducted under optimized conditions to determine the effectiveness of the predicted model. The experimental tannin content obtained under the optimized condition was 0.125 ± 0.02%. The resulting error between the predicted and experimental value was 2.4%. The predicted confidence interval (PI) (at a 95% confidence level) was (0.072; 0.173), which covered the values of tannin content obtained from the experiments, indicating a reliable model for predicting optimal conditions for the minimum tannin content under the SSF.

**Table 5:** Optimal process condition for minimum tannin content in cassava leaves.

Process condition	Carbon concentration (%)	Nitrogen concentration (%)	Moisture content (%)	Incubation time (h)	Tannin content (%)	
					predicted	Experimental
Optimum level	1.4	0.55	57	96	0.122	0.125

### 3.6 Changes in the chemical composition of cassava leaves during solid-state fermentation

In line with the purpose of this study, the chemical composition as a function of cassava leaves processing was also evaluated during the fermentation process. According to the chemical composition displayed in Table 6, the unfermented cassava leaves contain 10.08 ± 0.04% crude protein, 1.86 ± 0.01% total fat, 2.13 ± 0.01% ash, 17.90 ± 0.0% fibers, and 68.03 ± 0.03% carbohydrate. In the fermented cassava leaves, proteins, ash, and fats content increased with the increase in fermentation time while the content of crude fibers and carbohydrates decreased. Hence, after 96 h of fermentation with *S. cerevisiae*, the results showed that the content of proteins, ash, and fats the processed leaves increased by 16.07%, 4.73%, and 3.86%, respectively. These results are in well agreement with previously published results for the solid-state fermentation with *S. cerevisiae* of gari and flour (Oboh and Akindahunsi, 2003), cassava peels (Oboh, 2006), pineapple residue (Correia et al., 2007), cassava flour (Oboh and Elusiyan, 2007), and yam peels (Aruna et al., 2017) to enrich their of nutritional value. The results revealed that the protein content in pineapple residues increased from 6.4 to 22%.

**Table 6:** Chemical composition of unfermented and fermented cassava leaves (% dry weight basis).

Composition (%)	Unfermented cassava leaves	Fermented Cassava leaves with *S. cerevisiae*			
		24 h	48 h	72 h	96 h
Crude protein	10.08 ± 0.04	14.11 ± 0.06	14.87 ± 0.01	15.28 ± 0.01	16.07 ± 0.02
Ash content	2.13 ± 0.01	5.09 ± 0.01	5.29 ± 0.01	4.80 ± 0.02	4.73 ± 0.02
Fat content	1.86 ± 0.01	3.01 ± 0.02	3.36 ± 0.03	3.55 ± 0.01	3.86 ± 0.02
Crude fiber	17.90 ± 0.08	16.59 ± 0.04	16.80 ± 0.01	16.46 ± 0.02	16.35 ± 0.03
Carbohydrate	68.03 ± 0.03	61.20 ± 0.01	59.68 ± 0.04	59.91 ± 0.05	58.99 ± 0.03

Iyayi and Losel (2001) investigated the effect of SSF on the protein content of cassava peels using four fungal strains, namely *Mucor strictus*, *Rhizomucor miehei*, *Aspergillus niger*, and *S. cerevisiae*. After 12 days of fermentation, *S. cerevisiae* produced the highest protein content, corresponding to an increase of 66.74%.

The significant increase in protein content of fermented cassava products under SSF could be due to the capability of *S. cerevisiae* to produce extracellular enzymes (protein), as well as due to the enrichment of the fermentation medium with nitrogen (Oboh and Akindahunsi, 2003). In the study conducted by Bayitse et al. (2015), they observed similar trends for the cassava residue fermented by *Trichoderma pseudokoningii* under SSF with and without nitrogen supplementation of the fermentation medium although the improvement degree was not similar in both cases. Hence, the result showed an improvement in protein content of 12.5% with urea, as compared to 6.37% obtained without nitrogen supplementation.

At the end of fermentation (96 h), it was observed that almost half of the initial fat content of cassava leaves was increased. This is in line with the observation of Oboh and Akindahunsi (2003), which suggest that during fermentation, some microorganisms can produce microbial oil. Oboh and Elusiyan (2007) reported a significant increase in fat content from 2.9 to 8.0% in fermented cassava flour under SSF with *S. cerevisiae*.

On the other hand, the chemical composition results in Table 6 show a significant decrease in carbohydrate content from 68.03 to 58.99 % in fermented cassava leaves with *S. cerevisiae* indicates under SSF for 96 h. The decrease in carbohydrate content during fermentation could be explained by the ability of *S.cerevisiae* strain to hydrolyze complex carbohydrates into sugars that are used as a carbon source in the synthesis of high-protein microbial biomass (Akintomide and Antai, 2012).

In this study, the results showed that fermenting cassava leaves with *S. cerevisiae* is more effective in increasing the protein content when compared with the conventional methods, such as heating and boiling, used to process the cassava leaves, for which even the decrease in protein content was noticed. Hence, under the optimal SSF conditions, the protein content was increased by 59.42% while it decreased by 58% when the leaves were thermally treated by heating/boiling for 30 min (Ngudi et al., 2003). This significant decrease clearly shows that the application of heat has a negative impact on the nutritional value of the processed leaves due to the significant loss of protein content. In addition, the low nutritional value of the leaves is related to the loss of other nutrients, such as vitamins and sugars, when the leaves are subjected to thermal treatment in the presence of water, suitable conditions to remove all water-soluble molecules (Lancaster and Brooks, 1983; Aregheore, 2012).

### 3.7 Tannase production under SSF

This study, to the best of our knowledge, is the only substantial and reliable report of tannase production under SSF by *S. cerevisiae*. The results obtained for tannase production were achieved under the optimal conditions listed in Table 5, supplemented with 1% tannic acid and at different fermentation periods (24, 48, 72, and 96 h). Tannase enzyme activity of fermented cassava leaves (24–96 h) varied between 0.11 to 0.42 (U/gds), as shown in Table 7, with a maximum activity of 0.53 U/gds after 72 h of incubation. Liu et al. (2016) reported a high yield for tannase production of 12.26 U/gds using *Aspergillus spp*. UCP1284 under SSF and cashew bagasse as substrate. The SSF medium was supplemented with 2.0% tannic acid; the moisture content and fermentation time were of 40% and the 48 h, respectively. Sabu et al. (2006) also reported a maximum yield for tannase activity of 0.85 U/gds by SSF with *Lactobacillus sp.* ASR-S1 and using coffee husk as a substrate. The SSF medium was supplemented with 0.6% tannic acid; the moisture content and fermentation time were of 50% and 72 h, respectively.

**Table 7:** Tannase production under SSF.

Incubation time (h)	Tannase activity (U/gds)
24	0.11 ± 0.03
48	0.34 ± 0.01
72	0.53 ± 0.06
96	0.42 ± 0.04

## 4 Conclusions

In conclusion, this study focused on the reduction of the anti-nutrients level of cassava leaves by a simple, low cost, and energy-effective method, showed the effectiveness of solid-state fermentation with *S. cerevisiae* to reduce the tannin content of cassava leaves within the safe limits by optimizing the fermentation conditions. Moreover, compared to the conventional methods reported in the literature, the nutritional value was improved by the increased protein content. The present study also highlighted the capability of *S. cerevisiae* to produce extracellular tannase, one of the major enzymes used in industrial applications. Yet, further studies for optimization of the yield of tannase are necessary.

## Declarations

### Author contribution statement

Mohamed Hawashi: Conceived and designed the experiments; Performed the experiments; Analyzed and interpreted the data; Wrote the paper.

Ali Altway: Analyzed and interpreted the data.

Tri Widjaja: Conceived and designed the experiments; Contributed reagents, materials, analysis tools or data.

Setiyo Gunawan: Conceived and designed the experiments; Analyzed and interpreted the data; Contributed reagents, materials, analysis tools or data.

### Funding statement

This work was supported by the Ministry of Research, Technology and Higher Education of the Republic of Indonesia (grant no. 849/PKS/ITS/2018).

### Competing interest statement

The authors declare no conflict of interest.

### Additional information

No additional information is available for this paper.
